# Probing structural and dynamic properties of MAPbCl_3_ hybrid perovskite using Mn^2+^ EPR†

**DOI:** 10.1039/d4dt00116h

**Published:** 2024-04-03

**Authors:** Gediminas Usevičius, Justinas Turčak, Yuxuan Zhang, Andrea Eggeling, Žyginta Einorytė, Michael Allan Hope, Šarūnas Svirskas, Daniel Klose, Vidmantas Kalendra, Kestutis Aidas, Gunnar Jeschke, Jūras Banys, Mantas Šimėnas

**Affiliations:** a Faculty of Physics, Vilnius University Sauletekio 3 10257 Vilnius Lithuania mantas.simenas@ff.vu.lt; b Laboratory of Magnetic Resonance, Institute of Chemical Sciences and Engineering, École Polytechnique Fédérale de Lausanne (EPFL) Lausanne Switzerland; c Department of Chemistry and Applied Biosciences, ETH Zurich Vladimir-Prelog-Weg 1-5/10 8093 Zurich Switzerland

## Abstract

Hybrid methylammonium (MA) lead halide perovskites have emerged as materials exhibiting excellent photovoltaic performance related to their rich structural and dynamic properties. Here, we use multifrequency (X-, Q-, and W-band) electron paramagnetic resonance (EPR) spectroscopy of Mn^2+^ impurities in MAPbCl_3_ to probe the structural and dynamic properties of both the organic and inorganic sublattices of this compound. The temperature dependent continuous-wave (CW) EPR experiments reveal a sudden change of the Mn^2+^ spin Hamiltonian parameters at the phase transition to the ordered orthorhombic phase indicating its first-order character and significant slowing down of the MA cation reorientation. Pulsed EPR experiments are employed to measure the temperature dependences of the spin–lattice relaxation *T*_1_ and decoherence *T*_2_ times of the Mn^2+^ ions in the orthorhombic phase of MAPbCl_3_ revealing a coupling between the spin center and vibrations of the inorganic framework. Low-temperature electron spin echo envelope modulation (ESEEM) experiments of the protonated and deuterated MAPbCl_3_ analogues show the presence of quantum rotational tunneling of the ammonium groups, allowing to accurately probe their rotational energy landscape.

## Abstract 2

First comprehensive EPR study of Mn^2+^ ion incorporation in lead halide hybrid perovskites. We use these ions as local probes to study the structural and dynamic properties of MAPbCl_3_.

## Graphical abstract

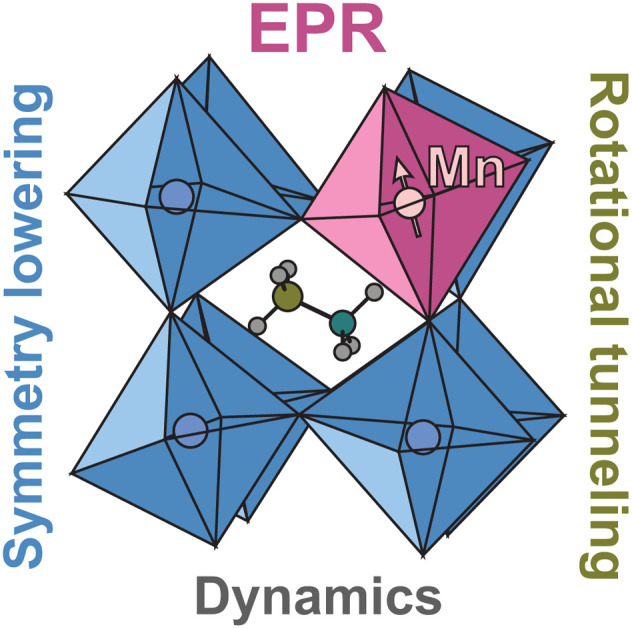

## 1. Introduction

In the last decade, hybrid methylammonium (CH_3_NH_3_^+^, MA) lead halide perovskites MAPbX_3_ (X = I, Br, Cl) and related compounds have emerged as highly promising materials for photovoltaic applications including high-performance solar cells,^1–4^ light-emitting diodes,^5^ and photodetectors.^6^ The power conversion efficiency of solar cells utilizing these compounds has undergone a remarkable increase and currently exceeds 25%.^3,4^ The high performance of MAPbX_3_ arises from the interplay of several physical factors including a large absorption coefficient,^7^ optimal band gap,^8^ long carrier diffusion length,^9^ low exciton binding energy,^10^ and defect tolerance.^11^ These properties are tightly linked to the rich structural and dynamic phenomena present in these materials, such as structural phase transitions and MA cation dynamics, which are currently under intense investigation.^8,11–18^

Among many other experimental techniques,^19^ electron paramagnetic resonance (EPR) spectroscopy has proved to be a very powerful tool for the investigation of phase transitions, long-range order and dynamics of classical inorganic (including CsPbX_3_ ^20,21^)^22–26^ and hybrid (mostly formate-based) perovskites.^27–38^ The main limitation of EPR is the requirement of unpaired electrons, so-called paramagnetic centers, to be present in the studied material. The majority of perovskite compounds intrinsically do not have such centers, and thus they have to be introduced by high-energy irradiation or by substituting diamagnetic ions with a low concentration of paramagnetic impurities such as transition metal ions (*e.g.* Mn^2+^), which then act as local structural probes enabling EPR experiments.^26,39^

However, a successful incorporation of paramagnetic centers in MAPbX_3_ seems to be challenging due to the phase separation and clustering tendencies of the impurities. For example, Kubicki *et al.*^40^ used NMR relaxation measurements to demonstrate that Co^2+^ is not incorporated into the perovskite lattice of MAPbI_3_. Náfrádi *et al.* used multifrequency continuous-wave (CW) EPR spectroscopy to study paramagnetic Mn^2+^ ions in MAPbI_3_ obtained by solvent synthesis.^41^ For 1 mol% substitution, the authors reported a broad EPR line with poorly resolved spectral signatures of Mn^2+^ ions and claimed a homogeneous distribution of these impurities in the crystal lattice. However, such a line broadening likely originated from substantial interactions between the paramagnetic ions indicating a highly inhomogeneous distribution and clustering of Mn^2+^ impurities in MAPbI_3_. Several other studies have claimed a successful incorporation of very large fractions of Mn and Cr in MAPbX_3_,^42–44^ but EPR spectra were not reported.

Here, inspired by the recent achievements of mechanosynthesis in lead halide perovskites,^40,45^ we show incorporation of a small amount of Mn^2+^ ions in MAPbCl_3_ hybrid perovskite using this synthesis method. We use a combined multifrequency CW and pulsed EPR approach to explore these paramagnetic ions in the crystal lattice. Our CW EPR experiments show a homogeneous distribution of these centers together with a superimposed broad EPR line, which is assigned to the clustered Mn^2+^ ions. We probe the local environment of the well-diluted impurities, which provides information on the MA cation dynamics and the structural phase transitions. The pulsed EPR experiments are used to measure the relaxation and decoherence properties of the Mn^2+^ spin centers revealing information on the lattice and molecular dynamics in the orthorhombic phase of MAPbCl_3_. In addition, low-temperature pulsed electron spin echo envelope modulation (ESEEM) experiments reveal field-independent signals originating from quantum rotational tunneling. By performing ESEEM experiments on deuterated analogues and comparing with density functional theory (DFT) calculations, we assign the tunneling to the NH_3_ groups of MA allowing us to precisely probe the complex energy landscape of these rotors in MAPbCl_3_.

## 2. Experimental and simulation details

### 2.1. Sample synthesis

CH_3_NH_3_PbCl_3_:Mn powder sample with a nominal Mn^2+^ concentration of 1 mol% was obtained by mechanosynthesis. MACl (54 mg, 0.8 mmol), PbCl_2_ (220 mg, 0.8 mmol), and MnCl_2_ (1.0 mg, 8 μmol) were ball-milled with a Retsch MM 400 in an agate grinding jar (10 mL) with one agate ball (∅ 10 mm) for 60 min at 25 Hz, before annealing the powder at 100 °C for 10 min. Deuterated CD_3_NH_3_PbCl_3_:1 Mn^2+^ mol% and CD_3_ND_3_PbCl_3_:1 Mn^2+^ mol% samples were prepared from CD_3_NH_3_Cl or CD_3_ND_3_Cl precursors. CD_3_NH_3_Cl was purchased from Sigma-Aldrich and used without further purification. CD_3_ND_3_Cl was prepared from CD_3_NH_3_Cl by dissolving in D_2_O (1 : 200 molar ratio), stirring for 2 days, and drying *in vacuo*. The obtained samples were stored at −80 °C or liquid nitrogen temperatures. Note that the 1 Mn^2+^ mol% concentration was chosen to provide a sufficiently intense EPR signal after screening several samples with different Mn^2+^ doping levels.

The synthesized CH_3_NH_3_PbCl_3_:1 Mn^2+^ mol% powder sample was characterized using powder X-ray diffraction (PXRD), Raman spectroscopy, and ^1^H NMR spectroscopy. The Raman spectrum was collected with Spectroscopy & Imaging GmbH Raman spectrometer. The 785 nm laser excitation was used for the measurements. The beam was focused with optical microscope onto the sample. ^1^H solid-state NMR spectra were recorded with a Hahn echo pulse sequence at room temperature and 50 kHz magic-angle spinning rate using an 11.7 T Bruker Avance III spectrometer and a 1.3 mm triple-resonance low-temperature magic-angle spinning probe.

### 2.2. EPR spectroscopy

The powder samples were placed into 4, 1.6 and 0.9 mm outer diameter EPR tubes for measurements at X- (∼9.5 GHz), Q- (∼34 GHz) and W-band (∼94 GHz) frequencies, respectively. The X- and Q-band EPR experiments were performed using a Bruker ELEXSYS E580/IF-Q EPR spectrometer equipped with Bruker ER4118X-MD5 (pulsed X-band), high-Q ER4102ST (CW X-band) and EN5107D2 (pulsed/CW Q-band) microwave resonators. To increase sensitivity of the pulsed EPR experiments at X-band, we used a probehead equipped with a cryogenic low-noise amplifier, as described in ref. 46. The W-band EPR experiments were performed using a Bruker ELEXSYS E680 X-/W-band spectrometer equipped with an EN 680-1021H resonator. Helium flow cryostats were used to stabilize the sample temperature.

The CW EPR experiments were performed at X- and Q-band frequencies using 3 G and 100 kHz (X-band), and 3 G and 50 kHz (Q-band) field modulation. The microwave power was adjusted at each temperature to avoid signal saturation. The CW EPR measurements with a P:Si reference sample of known spin concentration were performed at 25 K using 1 G field modulation.

For pulsed EPR experiments, we used 32 ns (X- and Q-band) and 80 ns (W-band) durations of the π-pulse. The echo-detected field sweep (EDFS) spectra were recorded using the Hahn echo pulse sequence (π/2–*τ*–π–*τ*–echo), with the interpulse delay *τ* set to 150 ns. The decoherence time *T*_2_ was obtained using the same pulse sequence with incrementing the interpulse delay *τ*. The longitudinal relaxation time *T*_1_ was obtained using the inversion recovery pulse sequence (π–*τ*′–π/2–*τ*–π–*τ*–echo), where the wait time *τ*′ was incremented until a full recovery of magnetization was observed as determined by integrating the full echo width. For the three-pulse (3p) ESEEM experiments, we used the π/2–*τ*–π/2–*τ*′–π/2–*τ*–echo pulse sequence, where the delay *τ*′ was incremented. For the Hahn echo measurements a two-step offset correction phase cycle was employed, while for the 3p ESEEM experiments we used a four-step phase cycle to cancel unwanted echoes.

The *T*_1_ values were determined by fitting a stretched exponential function *V* = *a*(1 − *b* exp(−(*τ*′/*T*_1_)^γ^)) to the inversion recovery data, which provided a significantly better agreement compared to single and bi-exponential functions. The decoherence time *T*_2_ was obtained by fitting a stretched exponential decay *V* = *a* exp(−(2*τ*′/*T*_2_)^γ^) to the Hahn echo data. To obtain the frequency spectra of the 3p ESEEM experiments, the time-domain data was divided by a fitted stretched-exponential decay function followed by subtraction of unity, apodization, zero-filling and fast Fourier transform with cross-term averaging.^47^

### 2.3. EPR simulation details

The CW EPR spectra were simulated using EasySpin 5.2.35^48^ running on MATLAB R2021b (The MathWorks Inc.).

The simulations of the 3p ESEEM signals of the NH_3_ group tunneling were performed by the density operator formalism using home-written MATLAB scripts, as described in our previous studies.^36,49,50^ The hyperfine interactions between the Mn^2+^ center and NH_3_ group protons were calculated using the point-dipole approximation from the low-temperature structure of MAPbCl_3_.^51^ Only the eight nearest MA cations to the Mn^2+^ center were considered in the simulations, as the effect from more distant cations was negligible. For each group, we simulated and multiplied time-domain traces corresponding to different orientations of the magnetic field, which is here justified due to negligible differences in the simulated 3p ESEEM patterns between both electron spin manifolds.^52^ The final 3p ESEEM signal was obtained by calculating the weighted average over all orientations followed by the weighted average over a Gaussian rotational barrier distribution of the NH_3_ group.^50^

The rotational barrier *V*_3_ of the NH_3_ group was calculated from the experimentally determined tunneling frequency *ν*_t_, defined as the energy difference between the two lowest-energy ro-librational substates,^53^ by diagonalizing the rotational Hamiltonian in a basis of the free quantum rotor.^54^ The rotational constant *B* = *ħ*^2^/2*I* of the NH_3_ group was calculated based on the moment of inertia *I* obtained from the optimized DFT structures. For a confined MA cation, we used *B* = 0.7604 meV and 0.7750 meV for the NH_3_ group situated further away and close to the Mn^2+^ center, respectively (see DFT calculation details). For the NH_3_ group in a free MA cation, we obtained *B* = 0.7688 meV. For the CH_3_ group, we used a typical value of 0.655 meV.

### 2.4. DFT calculations

All electronic structure calculations were performed using Gaussian 16.^55^ To model the rotational barriers of the MA cation, a single orthorhombic cell of MAPbCl_3_ was taken from the experimental crystal structure obtained at 80 K.^51^ The effect of manganese doping was accounted for by replacing one of the Pb^2+^ ions with a Mn^2+^ cation. Herein, two structures were constructed, where Mn^2+^ cation was located close to either ammonium or methyl moieties of the MA cation as shown in Fig. S1 (ESI†). To maintain full local coordination of the paramagnetic Mn^2+^ cation, three additional Cl – anions adjacent to Mn^2+^ were retained as well. The geometry of the MA cation was first fully optimized, while the structure of the framework around it was kept frozen. The geometry optimization was performed using a B3LYP exchange – correlation functional,^56,57^ the cc-pVTZ basis set^58^ for the MA cation, the aug-cc-pVDZ basis^59^ for the Cl – anions, and the LanL2DZ pseudopotential^60^ for the metal ions. An ultrafine integration grid was utilized as implemented in Gaussian 16. Our test calculations showed that this particular choice for the basis sets leads to well-converged results for the rotational barriers in a rather cost-effective manner. In addition, several exchange–correlation functionals including B3LYP, M06-2X,^61^ B97D,^62^ and wB97XD^63^ provided similar results for rotational barriers, where the computed values varied by about 10%. Hence, B3LYP functional was selected for electronic structure calculations in this work.

The potential energy surfaces were scanned for the rotation of either the methyl or the ammonium moiety around the C–N bond of MA using the same level of theory as for the geometry optimization. The internal degrees of freedom of the moiety being rotated were relaxed during the potential energy surface scan, while the geometry of the other group as well as the framework was kept fixed. The rotational barriers for an isolated MA cation were also calculated using the B3LYP functional and cc-pVTZ basis set. The rotational barriers were evaluated as the energy difference between the staggered and eclipsed conformations of MA.

## 3. Results and discussion

### 3.1. Initial sample characterization

Prior running the EPR experiments, we characterized the synthesized MAPbCl_3_:Mn sample using PXRD, Raman spectroscopy, and ^1^H NMR spectroscopy. The obtained PXRD pattern (Fig. S2a†) is typical for MAPbCl_3_ compound and reveals its cubic symmetry at room temperature. The measured room-temperature Raman spectrum (Fig. S2b†) also corresponds to MAPbCl_3_. We also obtained identical ^1^H solid-state NMR spectra of pure MAPbCl_3_ and Mn^2+^-doped MAPbCl_3_:Mn samples (Fig. S2c†). These observations indicate that introduction of 1 Mn^2+^ mol% does not alter the overall structure of MAPbCl_3_.

### 3.2. CW EPR

**Fig. 1:**
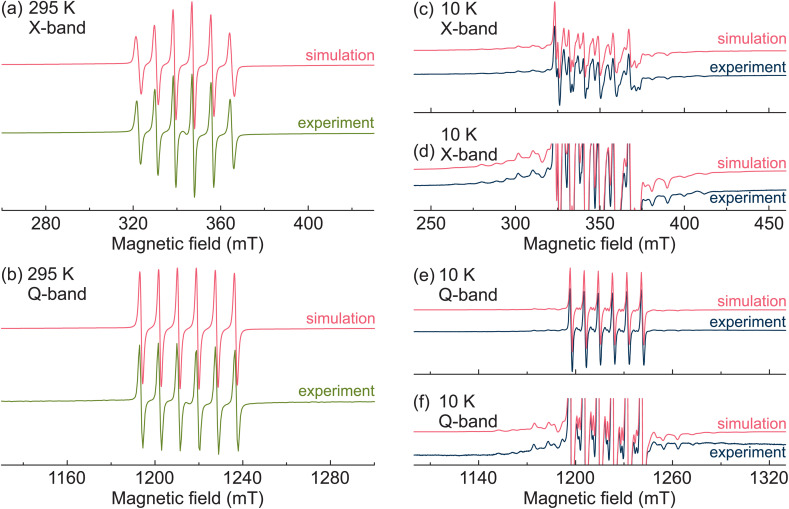
Normalized X- and Q-band CW EPR spectra of MAPbCl_3_:Mn measured at (a and b) room temperature and at (c–f) 10 K. (d) and (f) show emphasis on the outer fine structure transitions. The simulated spectra are presented in red.

First, we performed X- and Q-band CW EPR experiments to characterize the Mn^2+^ centers in MAPbCl_3_:Mn hybrid perovskite. The obtained room-temperature CW EPR spectra are presented in Fig. 1a and b revealing typical powder patterns of the Mn^2+^ ions in the 3d^5^ electronic configuration (^6^S_5/2_ ground state). The total electron spin of this state is *S* = 5/2, which results in five EPR transitions (Δ*m*_S_ = ±1, where *m*_S_ is the magnetic electron spin quantum number).^39,64^ For non-zero zero-field splitting (typical for lower-symmetry environments), the resonance fields of these transitions are different resulting in a fine structure in the EPR spectrum. The hyperfine interaction between the unpaired electrons and the nuclear spin *I* = 5/2 of the ^55^Mn isotope causes further splitting of each fine structure transition into six hyperfine lines (Δ*m*_S_ = ±1 and Δ*m*_I_ = 0).^39,64^

The room-temperature spectra of MAPbCl_3_:Mn are dominated by the well-resolved Mn hyperfine lines of the central fine structure transition (*m*_S_ = −1/2 ↔ 1/2). The outer transitions (*m*_S_ = ±3/2 ↔ ±1/2, *m*_S_ = ±5/2 ↔ ±3/2) are not visible (Fig. 1a and b) implying a negligible value of the zero-field splitting. We analyzed the observed spectra using spectral simulations based on the following spin Hamiltonian:^65^1
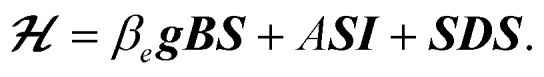


Here, the first term describes the electron Zeeman interaction characterized by the *g*-factor of the Mn^2+^ center. ***B*** and *β*_e_ are the external magnetic field and the Bohr magneton, respectively. The hyperfine interaction is described by the second term, where *A* is the isotropic hyperfine coupling constant. The last term takes into account the fine structure of the spectrum, where the fine structure tensor ***D*** can be parametrized by the axial *D* and the orthorhombic *E* zero-field splitting parameters.

The simulations of the room-temperature X- and Q-band CW EPR spectra of MAPbCl_3_:Mn are also presented in Fig. 1a and b revealing a good agreement with the experiments. The spin Hamiltonian parameters used for simulations at both frequency bands are *g* = 2.0009(1), *A* = −239(1) MHz, *D* = 0, and *E* = 0. The obtained value of the *g*-factor is typical for the Mn^2+^ centers, while the magnitude and sign of *A* reveals a Mn–Cl coordination bond.^66^ This indicates that the Mn^2+^ impurities substituted Pb^2+^ ions and formed MnCl_6_ octahedra confirming a successful incorporation of these paramagnetic centers in MAPbCl_3_. The obtained zero value of *D* reveals a cubic environment of the Mn^2+^ ions in MAPbCl_3_ in agreement with the cubic crystal symmetry of MAPbCl_3_ at this temperature.^67,68^

**Fig. 2:**
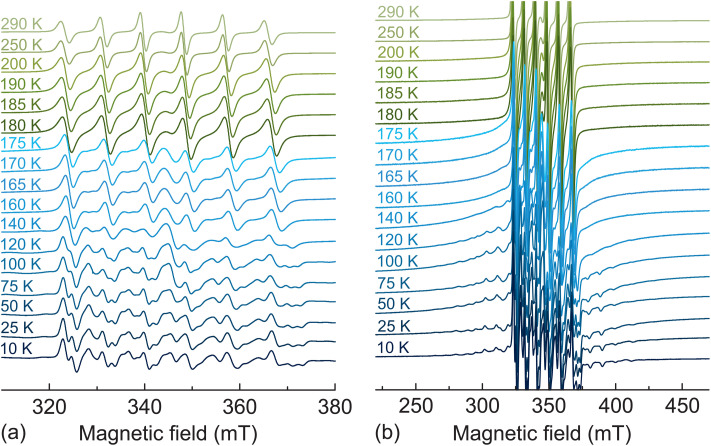
Normalized X-band EPR spectra of MAPbCl_3_:Mn recorded at different temperatures. Emphasis on the (a) central and (b) outer transitions.

We further performed temperature dependent CW EPR experiments to investigate, whether the Mn^2+^ centers are sensitive to the structural phase transitions in MAPbCl_3_, which in the pure compound occur at 179 K (cubic-tetragonal) and 173 K (tetragonal-orthorhombic).^67,68^ The recorded X-band (Fig. 2) EPR spectra reveal only minor spectral changes upon cooling down to about 175 K, while at this temperature the spectrum experiences a drastic change, as the outer fine structure transitions start to appear and become resolved at about 140 K. This indicates a sudden increase of the fine structure parameters. The same behavior is observed in the Q-band spectra (Fig. S3†), where the spectrum experiences a drastic change at slightly lower temperature of 170–175 K. A small difference in temperature can be explained by different probeheads used to run the X- and Q-band EPR experiments.

We expect that this change at 175 K corresponds to the tetragonal-orthorhombic phase transition rather than the cubic-tetraognal symmetry lowering, which is expected to have a less pronounced effect on the lattice distortion. This behavior confirms a successful incorporation of the Mn^2+^ ions in MAPbCl_3_ and their susceptibility to the significant symmetry change occurring at the phase transition. Note that the cubic-tetragonal phase transition in MAPbX_3_ is also poorly resolved using some other experimental methods such as dielectric spectroscopy^11,17^ and quasielastic neutron scattering.^69^

Upon further cooling, the outer fine structure lines move away from the central transition indicating a further increase of the zero-field splitting parameters (Fig. 2b and S3b†). The simulated X- and Q-band EPR spectra obtained at the lowest measured temperature (10 K) are also presented in Fig. 1 showing a good agreement with the experimental data. A small disagreement in the region of the central transition likely occurs due to the nuclear quadrupole interaction of ^55^Mn nucleus, which is ignored in our simulations. The obtained simulation parameters are *g* = 2.0004(1), *A* = −245(1) MHz, *D* = 434(10) MHz, and *E* = 63(5) MHz. The non-zero values of *D* and *E* parameters reveal a substantial rhombic distortion of the MnCl_6_ octahedra in agreement with the orthorhombic crystal symmetry of MAPbCl_3_ at low temperature. The simulations of the spectra obtained at other selected temperatures are presented in Fig. S4 and S5† showing a good agreement with the experiment despite a broad distribution of the zero-field splitting parameters in the low-temperature phase.

**Fig. 3:**
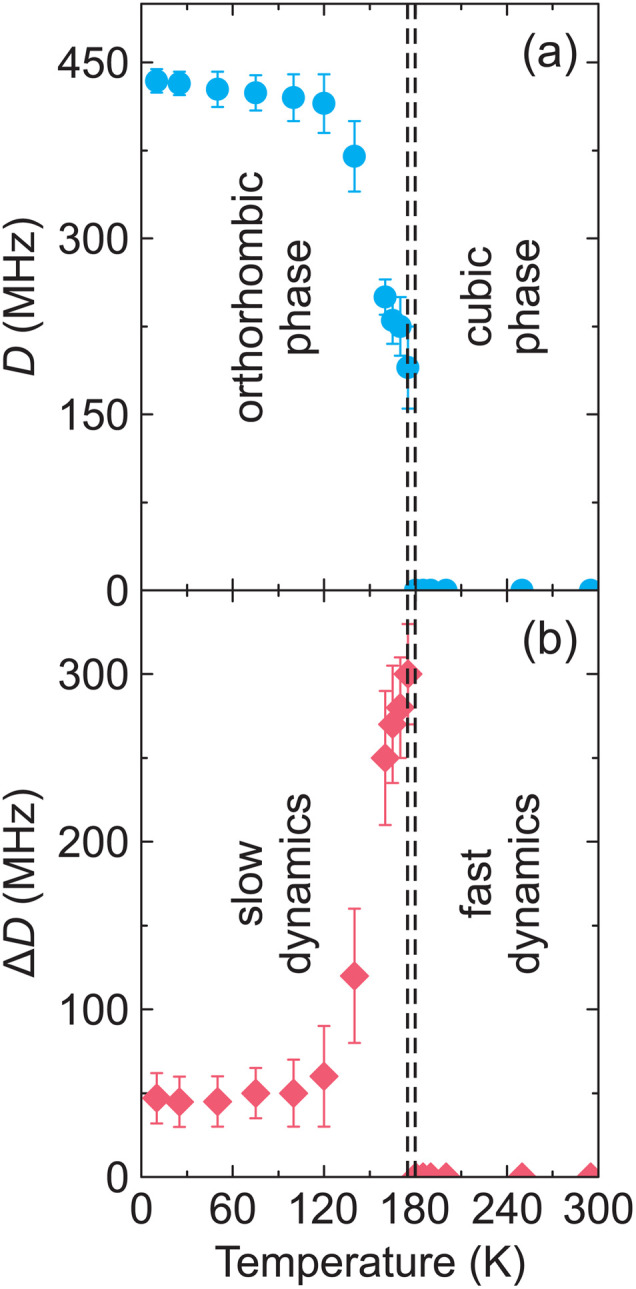
Temperature dependence of the (a) axial zero-field splitting parameter *D* and (b) its distribution Δ*D* as obtained from the simulations of the X- and Q-band EPR spectra of MAPbCl_3_:Mn. The dashed lines indicate the temperatures of the phase transitions in MAPbCl_3_.

Fig. 3a shows the temperature dependence of the axial zero-field splitting parameter *D* as obtained from the simulations revealing its gradual increase upon cooling in the orthorhombic phase, which is a typical phase transition behavior.^26,39^ The obtained dependence is less abrupt than observed for the transitions in the related [(CH_3_)_2_NH_2_][Zn(HCOO)_3_]^27^ and [(CH_3_)_2_NH_2_][Cd(N_3_)_3_]^35^ hybrid perovskites, which exhibit strong first-order (discontinuous) phase transitions occurring in very narrow temperature intervals (∼1 K). On the other hand, it is more abrupt compared to the second-order (continuous) phase transition obtained for the [NH_4_][Zn(HCOO)_3_] compound and covering more than 100 K temperature range,^37^ which indicates that the phase transition in MAPbCl_3_ is likely of the weak first-order type. Note that an attempt to approximate the determined temperature dependence of *D* using a power law^37^ failed further supporting first-order character of the transition. The first-order phase transitions in MAPbX_3_ compounds were also proposed based on other experimental techniques including calorimetric and dielectric experiments.^67,68^

To obtain a good agreement between the measured and simulated spectra in the orthorhombic phase, we had to use a substantial *D* parameter distribution Δ*D* (defined as the full width at half maximum (FWHM) of a Gaussian distribution). The temperature dependence of Δ*D* is presented in Fig. 3b revealing a sudden increase at the phase transition point. We relate such a behavior to an abrupt slowing down of the dynamics of the inorganic framework influenced by the MA cation ordering.^11,17^ In the disordered cubic phase, the MA cations are known to exhibit very fast rotation on the ps or faster time scale,^18^ which is much faster than the EPR measurement frequency (ns scale). Thus, the anisotropic fine structure tensor is completely averaged out together with its distribution, *i.e. D* = 0 and Δ*D* = 0. In the orthorhombic ordered phase, the time scale of the framework dynamics becomes slower than that of the EPR resulting in *D* > 0 and Δ*D* > 0. The decrease of Δ*D* > 0 with further lowering of temperature reflects increased ordering of the system (reduction of different Mn^2+^ ion environments) indicating that the long-range order is only fully established below100 K (Fig. 3b) and not immediately at the phase transition. This is the same temperature at which the *D* parameter attains its asymptotic limit (Fig. 3a). Note that several groups reported onset of the three-fold MA cation rotation above 80 K in MAPbI_3_.^69^ The same effect can be expected in the chloride analogue, which may explain the observed temperature dependence of *D* in the orthorhombic phase.

The presence of non-zero Δ*D* at the lowest measured temperatures also indicates a non-uniform environment of the MnCl_6_ octahedra. It may originate from the crystal defects or may suggest that the ordering is not ideal in this system likely due to the soft-nature^70,71^ of the framework. Note that in the related formate-based perovskites containing dimethylammonium (DMA) cations, a broad distribution of the *D* parameter was observed even in the high-temperature disordered phases due to lower crystal symmetry and significantly slower DMA and framework dynamics.^27,35,72^

We also note that to obtain the best agreement between the simulated and experimental spectra, we had to take into account a broad EPR line (∼60 mT linewidth) centered at *g* = 2.0 (see Fig. S6†). We assign this line to regions of high local Mn^2+^ concentration suggesting clustering tendencies of these ions in MAPbCl_3_. Ignoring the possible spin exchange effects, our spectral simulations indicate that such clustered regions have about 10× more spins compared to the diluted Mn^2+^ species. A separate study is envisioned in future to investigate the formation of such regions.

We also performed CW EPR measurements at 25 K with a P:Si sample of a known spin concentration to determine the actual Mn^2+^ doping level in MAPbCl_3_:Mn. The measured Mn^2+^ concentration is ∼0.6 mol%, which is very close to the nominal concentration of 1 mol% expected for this sample. Note that we used both well-resolved and broad EPR signals to calculate the spin concentration indicating that the concentration of the diluted Mn^2+^ species is about 0.06 mol%.

### 3.3. Relaxation and decoherence

We used pulsed EPR experiments to further investigate the orthorhombic phase of MAPbCl_3_. First, we measured the X- and Q-band EDFS EPR spectra of MAPbCl_3_:Mn at 8 K (see Fig. S7†), which revealed a typical Mn^2+^ pattern in agreement with the CW EPR.

We further studied the relaxation and decoherence properties of the well-resolved Mn^2+^ centers in MAPbCl_3_. The spin–lattice relaxation time *T*_1_ was obtained by fitting a stretched exponential to the experimental X- and Q-band inversion recovery data (see Fig. S8 and S9†). To obtain the best fits, we had to monotonously increase the stretching factor *γ* from 0.33 at 5 K to 0.86 at 170 K indicating a nearly Gaussian distribution of the relaxation times.^73^

**Fig. 4:**
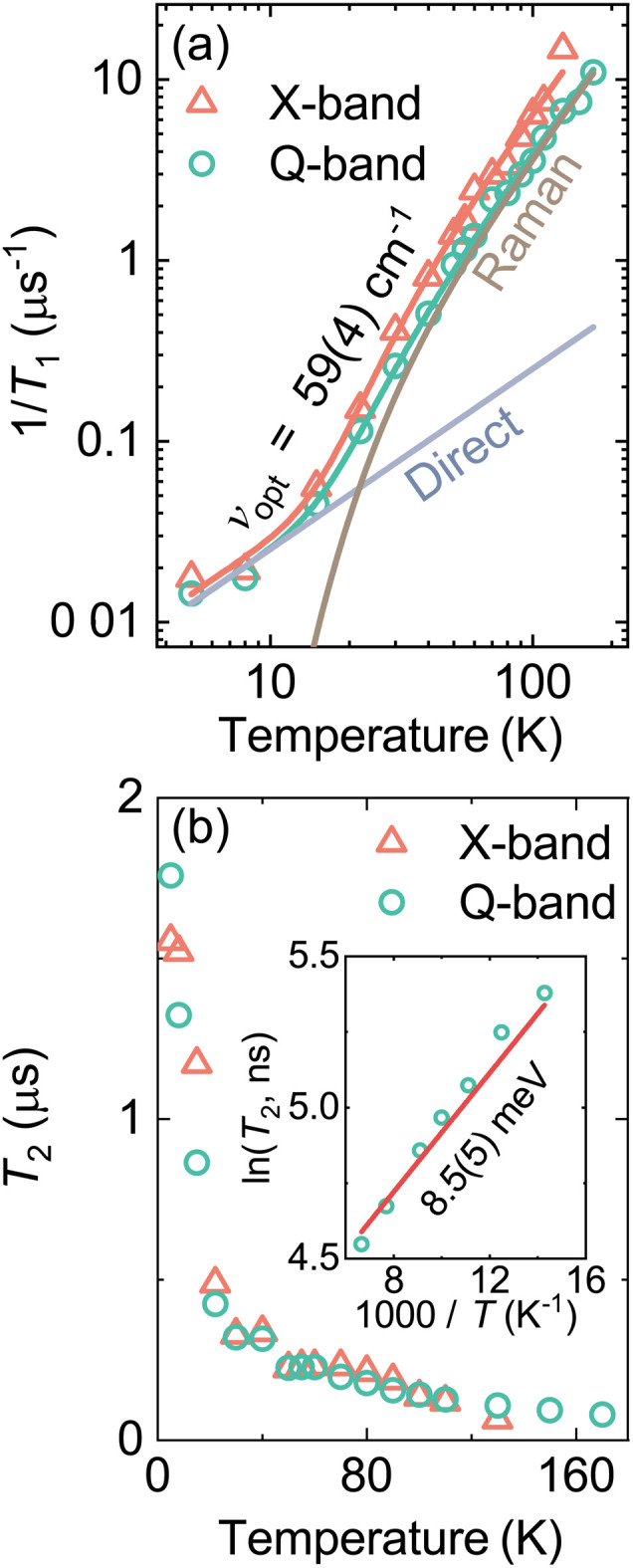
Temperature dependence of the (a) spin-lattice relaxation rate 1/*T*_1_ and (b) electron spin coherence time *T*_2_ of MAPbCl_3_:Mn obtained at X- (324.9 mT) and Q-band (1196.7 mT) frequencies. Solid curves in (a) represent the best global fit to the *T*_1_ data using a combination of the direct and Raman relaxation processes (eqn (2)) Separate contributions to the Q-band fit are also indicated in (a). Inset in (b) shows an Arrhenius plot of the *T*_2_ data.

The temperature dependence of the relaxation rate *T*_1_^−1^ is presented in Fig. 4a showing a gradual increase with increasing temperature. The experimental data was approximated using the following equation:^74^

2 *T*_1_^−1^ = *AT* + *B*csch^2^(*hν*_opt_/2*k*_B_*T*)

where the first term describes a direct (one-phonon) relaxation process due to the interaction with acoustic lattice phonons. The second term takes into account a two-phonon Raman process which involves spin coupling to an optical phonon branch of frequency *ν*_opt_. *A* and *B* are constants. Note that this relaxation model was previously successfully applied to describe various systems exhibiting phase transitions^74^ including formate-based hybrid perovskites.^30,32^ The simultaneous best fit to the X- and Q-band experimental data resulted in *ν*_opt_ = 59(4) cm^−1^. This value falls within the frequency band of the lattice phonons associated with the octahedral vibrations in the MAPbX_3_ systems^71^ indicating that these vibrations are driving the spin–lattice relaxation. Based on our previous works on formate-based perovskites,^30,32^ at the transition temperature, we may expect an increase in the *T*_1_ time (dip in relaxation rate) due to the coupling of the transition driving mode with the optical phonon responsible for the spin relaxation. However, due to too fast relaxation, we were not able to measure the relaxation properties at the phase transition point.

The temperature dependence of the coherence time *T*_2_ of the Mn^2+^ centers in MAPbCl_3_ is presented in Fig. 4b revealing a monotonic decrease with increasing temperature (see Fig. S10 and S11† for the Hahn echo decay data). A similar dependence and similar *T*_2_ values are observed at X- and Q-band frequencies. The value of *T*_2_ exhibits a rather fast decrease with temperature from about 1.5 μs at 5 K to about 0.5 μs at 20 K, while at higher temperature (>20 K), the decay is significantly less pronounced. Note that in our previous works, we also observed a similar kink in the *T*_2_ temperature dependence of the Mn^2+^ impurities in some formate-based hybrid perovskites containing different molecular cations.^30,37^ Since the framework of MAPbCl_3_ is purely inorganic, we can safely assume that the origin of this kink is unrelated to the change of the organic linker (*e.g.* formate) dynamics. Thus, based on the rotation barriers (see below), we tentatively assign this behavior to the onset of the stochastic methyl group reorientation with increasing temperature.

As indicated in the inset of Fig. 4b, the coherence time *T*_2_ exhibits the Arrhenius-type behaviour above ∼70 K, which can be described using the following equation:^30^

3 *T*_2_ = *T*_2,∞_*e*^−*E*_a_/*k*_B_*T*^

where *E*_a_ and *T*_2,∞_ are the activation energy and decoherence time at infinite temperature, respectively. The best fit for the Q-band data revealed *E*_a_ = 8.5(5) meV or 69(3) cm^−1^, which is close to the phonon frequency obtained from the *T*_1_ data suggesting that above 70 K the decoherence is tightly related to the vibrations of the inorganic framework. This is not surprising given that approximately in this temperature region the value of *T*_2_ becomes very close to *T*_1_ (see Fig. S12†). We also note that Leguy *et al.*^12^ observed molecular cation dynamics with similar energy scale in MAPbI_3_ using quasielastic neutron scattering. In light of our findings, this may indicate a tight coupling between the inorganic and organic sublattices in these compounds.

### 3.4. Quantum rotor tunneling

The methyl and ammonium groups act as hindered quantum rotors at low temperature exhibiting quantum rotational tunneling.^53,54^ The tunnel frequency *ν*_t_, defined as the splitting between the ro-librational ground states, is extremely sensitive to the rotational barrier *V*_3_ of the quantum rotor. This makes the rotational tunneling effect a unique and highly sensitive probe to study the local methyl and ammonium group environment.^75,76^ Recently, we observed and studied the methyl group tunneling using 3p ESEEM spectroscopy in DMA-based hybrid perovskites doped with paramagnetic Mn^2+^ and Co^2+^ ions.^36,77^ In contrast to ordinary ESEEM used to measure small hyperfine interactions with nearby nuclei, a distinct feature of the tunneling ESEEM is its independence of the magnetic field.^36^

**Fig. 5:**
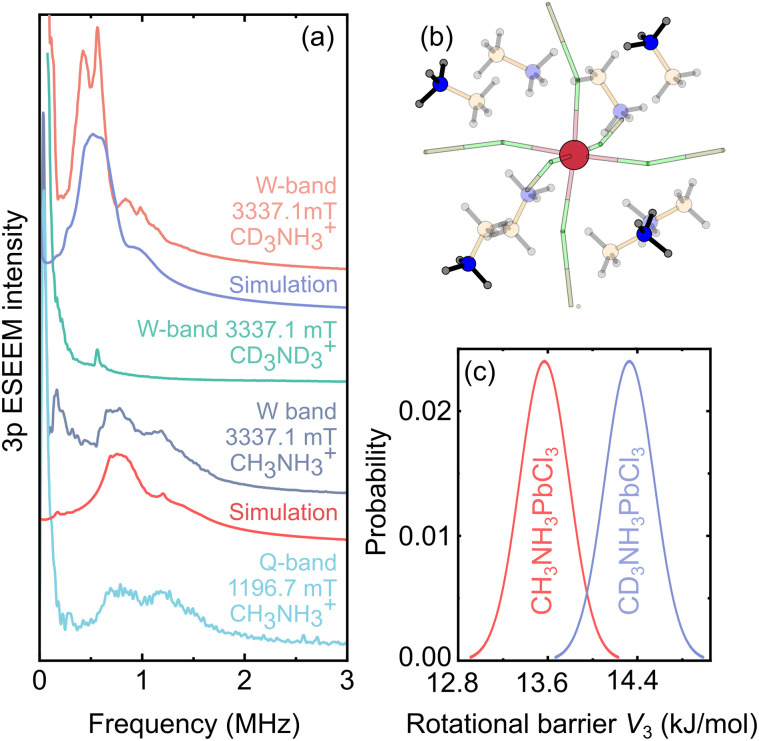
(a) Q- and W-band 3p ESEEM spectra of protonated and deuterated MAPbCl_3_:Mn compounds obtained at 8 K. The simulated tunneling ESEEM signals of the NH_3_ groups are presented below the experimental traces. (b) Structural model used to simulate the tunneling ESEEM. The highlighted NH_3_ groups were included in the simulation. (c) Distribution of the rotational barriers used to simulate the tunneling ESEEM spectra in (a).

The MAPbCl_3_:Mn system constitutes a very interesting case to study this effect, as the MA cation contains both methyl and ammonium rotors with different rotational barriers due to the latter group forming H-bonds with the inorganic framework. Thus, we attempted to observe the tunneling ESEEM of both groups in MAPbCl_3_ using Mn^2+^ probe ions. The Q- and W-band 3p ESEEM spectra of MAPbCl_3_:Mn obtained at 8 K are presented in Fig. 5a revealing two lines centered at about 0.95 MHz, which are independent of the magnetic field and thus can be assigned to tunneling dynamics (see Fig. S13† for the time-domain data). This is further supported by the W-band 3p ESEEM experiments of a fully deuterated CD_3_ND_3_PbCl_3_:Mn sample showing absence of these signals (Fig. 5a).

To identify which group is responsible for the observed tunneling ESEEM, we performed measurements of a partially deuterated CD_3_NH_3_ PbCl_3_:Mn compound. The obtained W-band spectrum (Fig. 5a) also shows the presence of two lines indicating that the tunneling signal originates from the NH_3_ group. However, the observed signals are shifted to lower frequency by about 0.5 MHz compared to the protonated analogue. This behavior may originate from a small shift of the MA position in the inorganic cage upon deuteration, which was also observed in other lead halide hybrid perovskites.^78^ Alternatively, the shift in frequency may also occur due to a small rotor-rotor coupling of the NH_3_ and CD_3_ groups,^49^ as the tunnel splittings of both groups become comparable upon methyl group deuteration. We also note that the fully deuterated CD_3_ND_3_PbCl_3_:Mn compound also shows a small signal in this frequency region (Fig. 5a), which likely originates from the incomplete deuteration level of the NH_3_ groups in this sample.

We further support our experimental results with DFT calculations based on two structural settings, where the Mn^2+^ ion is close to either the CH_3_ or NH_3_ group of the MA cation (see Fig. S1†). The obtained rotational barriers *V*_3_ of both groups in both structures are summarized in Table 1 together with the corresponding calculated tunneling frequencies *ν*_t_ (see EPR simulation details). Our DFT results indicate that the observed tunneling ESEEM originates from the ammonium groups, which are further away from the Mn^2+^ defect, as the calculated value of *ν*_t_ for these species is close to the measured ESEEM frequency. Note that the DFT calculations also show that the tunneling signal of the CH_3_ groups in MAPbCl_3_ should appear at substantially higher frequencies, which should result in very small modulation depths^36^ for these species further supporting our assignment of the observed tunneling ESEEM to the ammonium groups.

**Table 1:** Calculated rotational barriers *V*^DFT^_3_ and corresponding tunneling frequencies *ν*^DFT^_*t*_ of CH_3_ and NH_3_ groups for a free and confined MA cation. The measured rotational barrier *V*^exp^_3_ is also indicated

DFT structure	Rotor	*V* ^DFT^_3_ (kJ mol^−1^)	*ν* ^DFT^_t_ (MHz)	*V* ^exp^_3_ (kJ mol^−1^)	*ν* ^exp^_t_ (MHz)
Free MA	CH_3_	9.27	5.1	—	—
	NH_3_	9.27	18.2	—	—
CH_3_ close to Mn center	CH_3_	4.86	263.3	—	—
	NH_3_	13.10	1.3	13.57	1.15
NH_3_ close to Mn center	CH_3_	9.07	5.9	—	—
	NH_3_	17.91	0.1	—	—

Our DFT calculations also indicate that the ammonium group situated near the Mn^2+^ center has a significantly higher barrier of rotation, which translates to a very low tunneling frequency (Table 1). These species might be responsible for the measured low frequency (<0.1 MHz) ESEEM lines, though the intensity of such signals is also highly sensitive to the employed background correction.

To obtain a more quantitative comparison with the experiment, we performed simulations of the tunneling ESEEM based on the density operator formalism (see EPR simulation details). In our simulations, we included four more distant NH_3_ groups to the Mn^2+^ center from the eight nearest MA cations (Fig. 5b), as, based on the DFT calculations, the four nearest NH_3_ groups are expected to have substantially higher rotation barriers (see Table 1). This is also supported by our simulations, which yield significantly higher 3p ESEEM modulation depth than observed in the experiment, if the four nearest groups are included. The simulations also show that ammonium groups from other more distant MA cations have a negligible contribution to the tunneling ESEEM, and thus they have not been considered.

First, we attempted to simulate the measured data of the protonated MAPbCl_3_:Mn compound using a single value of the tunneling frequency. Our simulations proved unsuccessful, as the obtained ESEEM spectrum was significantly narrower compared to the experiment (Fig. S14†), which points to a substantial distribution for the tunneling frequency. To account for this, we assumed a Gaussian distribution of the rotation barrier and explored a vast space of the distribution parameters. The best agreement between the experiment and simulation (Fig. 5a) was obtained using a mean value of *V*_3_ = 13.57 kJ mol^−1^ (tunneling frequency *ν*_t_ = 1.15 MHz) and the FWHM of 0.22 kJ mol^−1^ (see Fig. 5c and Table 1). A small discrepancy between the simulation and experiment may originate from the simplified distribution and unaccounted effects of the Mn^2+^ center on the local lattice deformation. Note that Li *et al.*^79^ reported a similar energy barrier (∼12 kJ mol^−1^) required to rotate the H-bonded NH_3_ group in MAPbI_3_.

The simulation assuming absence of the rotor-rotor coupling of the partially deuterated CD_3_NH_3_PbCl_3_:Mn sample also required a distribution of the NH_3_ rotational barrier. The best agreement with the experiment was obtained using *V*_3_ = 14.36 kJ mol^−1^ (*ν*_t_ = 0.60 MHz) and a FWHM of 0.22 kJ mol^−1^ (see Fig. 5a and c). The mean values of the determined distributions in both compounds are rather close indicating that the associated structural changes due to partial methyl group deuteration do not significantly affect the H-bonds of the MA cations with the inorganic framework. However, as discussed above, we cannot exclude the possibility that the shift in ESEEM frequency is affected by the rotor–rotor coupling, and thus the determined distribution should be interpreted with care.

Finally, we also performed temperature dependent measurements of the tunneling ESEEM of the protonated sample, which revealed that the tunneling signal starts to shift to lower frequencies above 20 K and vanishes at about 30 K (see Fig. S15†). This is another signature for the quantum rotor tunneling, as normal nuclear ESEEM typically does not exhibit such a temperature dependence. The origin of this behavior may be interaction with phonons, which modulate the orientation of the rotational potential in the crystal lattice.^53^ On the other hand, several studies^69,80^ have found that in this temperature region, the MA cation starts to exhibit a classical reorientation around the C–N axis, which would also cause the disappearance of the tunneling signal. Note that the vanishing of the tunneling ESEEM corresponds to the change of slope in the temperature dependence of the *T*_2_ plot (Fig. 4b) suggesting that the spin decoherence is indeed affected by the change in the rotor dynamics.

## 4. Summary and conclusions

In this work, we probed the structural and dynamic properties of MAPbCl_3_ hybrid perovskite using multifrequency Mn^2+^ EPR spectroscopy. The CW EPR results demonstrated that mechanosynthesis is a suitable approach for incorporation of paramagnetic impurities in lead halide hybrid perovskites. Our results showed that the Mn^2+^ centers successfully substituted lead in the inorganic framework and formed MnCl_6_ octahedra. The temperature dependence of the CW EPR spectrum revealed that the diluted Mn^2+^ ions are highly sensitive to the tetragonal-orthorhombic phase transition and slowing down of the coupled MA and framework dynamics upon cooling.

The pulsed EPR measurements of MAPbCl_3_:Mn revealed a gradual increase of the *T*_1_ relaxation time on cooling, which can be described by a combination of direct and Raman processes. The latter mechanism is found to involve an optical phonon branch typical for compounds exhibiting structural phase transitions. The temperature dependence of the coherence time *T*_2_ of the Mn^2+^ ions was found to exhibit a kink at about 20 K, which was assigned to the slowing down of the stochastic methylgroup rotation with decreasing temperature.

The low-temperature 3p ESEEM experiments revealed signals independent of the external magnetic field, which originate from rotational tunneling of quantum rotors. The measurements of deuterated samples and DFT computations allowed us to assign these signals to the ammonium groups of the MA cations. This is the first observation of tunneling ESEEM for the ammonium group and for a molecular cation other than DMA.

Our experiments allowed us to probe the local environment of NH_3_ groups in the presence of the paramagnetic Mn^2+^ defect. For groups further away from this center, we determined the rotation barrier distribution with a mean value of 13.57 kJ mol^−1^ and a FWHM of 0.22 kJ mol^−1^. For the partially deuterated CD_3_NH_3_PbCl_3_ compound, the tunneling signal shifted to lower frequencies indicating a small increase in the rotational barrier, which may originate from a change of the MA cation position upon deuteration or a rotor–rotor coupling between the NH_3_ and CD_3_ groups. Our results demonstrates the capacity of tunneling ESEEM as a spectroscopic tool to precisely probe the local environment of a quantum rotor.

We also observed a substantial amount of Mn^2+^ ions clustered in the regions of high local concentration. A further study is envisioned to study, whether such a clustering is a synthesis effect or occurs during the post-synthesis sample treatment.

Note that we also attempted the same Mn^2+^ ion incorporation procedure in MAPbI_3_, but the obtained EPR spectrum indicated unsuccessful doping (see Fig. S16†). This suggest that a different incorporation approach might be necessary for the iodide analogue.^81^ Nevertheless, due to structural and dynamic similarities between the MAPbX_3_ compounds,^11,67^ we expect that our EPR results obtained for the MAPbCl_3_ analogue can be used to gain insights for the whole MAPbX_3_ family.

In general, our work is the first comprehensive EPR study of paramagnetic defect centers in lead halide hybrid perovskites. We anticipate that it will open new research avenues for EPR in this field yielding an important atomistic picture on the incorporated paramagnetic defect centers as well as on the structural and dynamic phenomena occurring in these materials. Our work is also highly relevant in the context of recent utilization of paramagnetic optically active defect centers (*e.g.* Yb^3+^) in lead halide perovskites for improved photovoltaic and optical properties.^45,82^

## Author contributions

Conceptualization: G.U., M.Š. Data curation: G.U., A.E., D.K., K.A., M.Š. Formal analysis: G.U., J.T., Ž.E., M.Š. Funding acquisition: K.A., G.J., J.B., M.Š. Investigation: G.U., J.T., M.A.H., A.E., Š.S., Ž.E., Y.Z., M.Š. Methodology: G.U., K.A., M.Š. Project administration: M.Š. Resources: M.A.H., Y.Z., V.K., G.J., J.B., M.Š. Software: G.U., J.T., Ž.E., G.J. Supervision: D.K., K.A., G.J., J.B., M.Š. Validation: A.E., K.A., G.J., M.Š. Visualization: G.U., M.Š. Writing – original draft: G.U., M.Š. Writing – review and editing: all authors.

## Conflicts of interest

There are no conflicts to declare.

## Supplementary Material

DT-053-D4DT00116H-s001
