# Supplementary material for: Probing structural and dynamic properties of MAPbCl3 hybrid perovskite using Mn2+ EPR
Source: Dalton Trans. 2024 Apr 3;53(17):7292–302. doi: 10.1039/d4dt00116h (PMC11059044; doi:10.1039/d4dt00116h)
Supplement: DT-053-D4DT00116H-s001 [file DT-053-D4DT00116H-s001.pdf]

Electronic Supplementary Material (ESI) for Dalton Transactions.
This journal is © The Royal Society of Chemistry 2024

Supplementary information

Probing structural and dynamic properties of $MAPbCl_3$ hybrid perovskite using Mn^{2+} EPR

Gediminas Usevičius, Justinas Turčak, Yuxuan Zhang, Andrea Eggeling, Žyginta Einorytė, Michael Allan Hope, Šarūnas Svirskas, Daniel Klose, Vidmantas Kalendra, Kęstutis Aidas, Gunnar Jeschke, Jūras Banys, and Mantas Šimėnas*

E-mail: mantas.simenas@ff.vu.lt

Additional DFT data

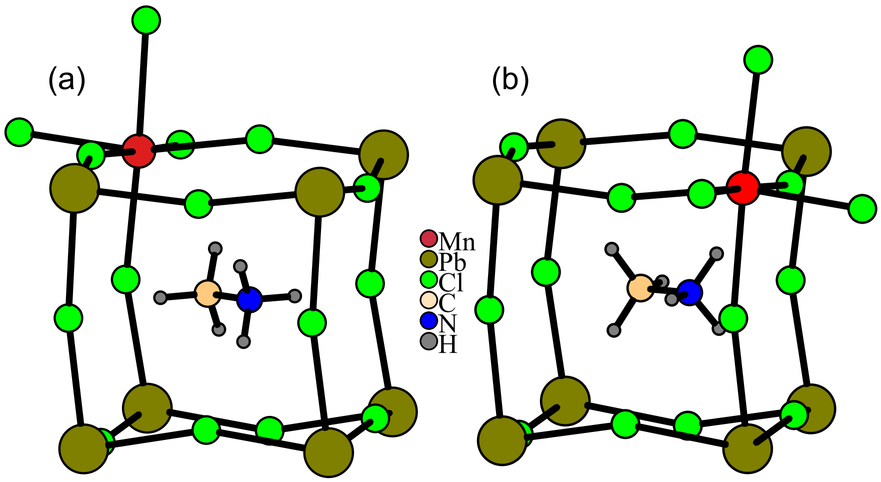

Figure S1: DFT structure of $MAPbCl_3$:Mn used for calculation of the rotation potentials, when the Mn^{2+} ion is close to the (a) methyl and (b) ammonium group of the MA cation.

Initial sample characterization

The room-temperature PXRD pattern of the $MAPbCl_3$:Mn sample reveals that this compound has a cubic symmetry at room temperature (Figure S2a). The obtained pattern is in a good agreement with that of the pure $MAPbCl_3$ compound.[1]

The room-temperature Raman spectrum of the $MAPbCl_3$:Mn sample is presented in Figure S2b showing Raman bands originating from MA cations. The measured spectrum corresponds to the spectrum of the undoped $MAPbCl_3$ compound as reported in Ref. 2.

The room-temperature ^{1}H solid-state NMR spectrum of $MAPbCl_3$:Mn is presented in Figure S2c. The obtained spectrum is identical to that of the pure $MAPbCl_3$.

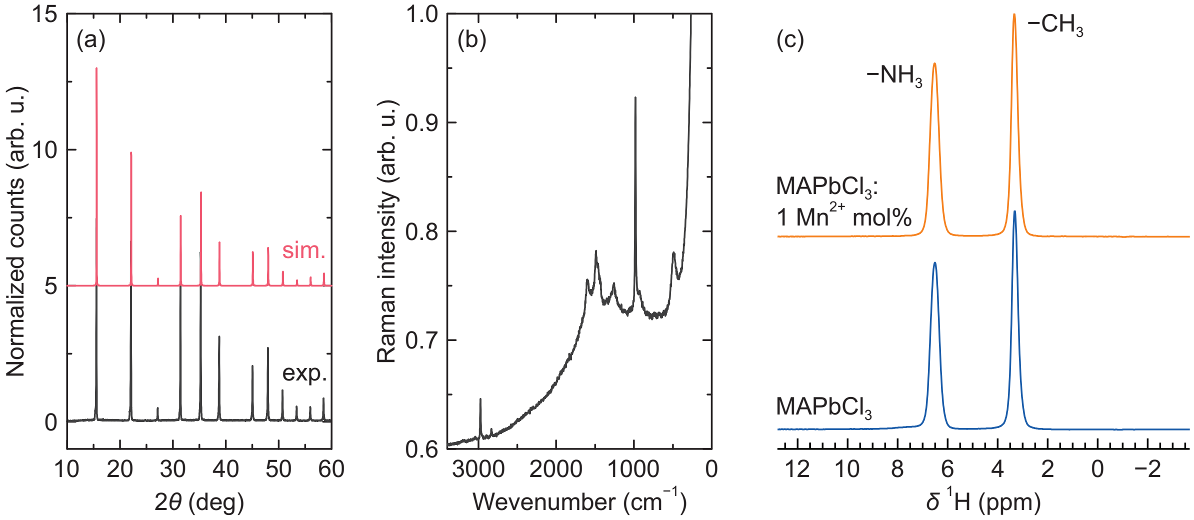

Figure S2: (a) Room-temperature PXRD of $MAPbCl_3$:Mn. The simulated spectrum is presented for comparison. (b) Room-temperature Raman spectrum of $MAPbCl_3$:Mn. (c) ^{1}H NMR spectra of $MAPbCl_3$:Mn and undoped $MAPbCl_3$ obtained at room temperature, 11.7 T, 40 s recycle delay, and 50 kHz magic-angle spinning rate.

Additional EPR data

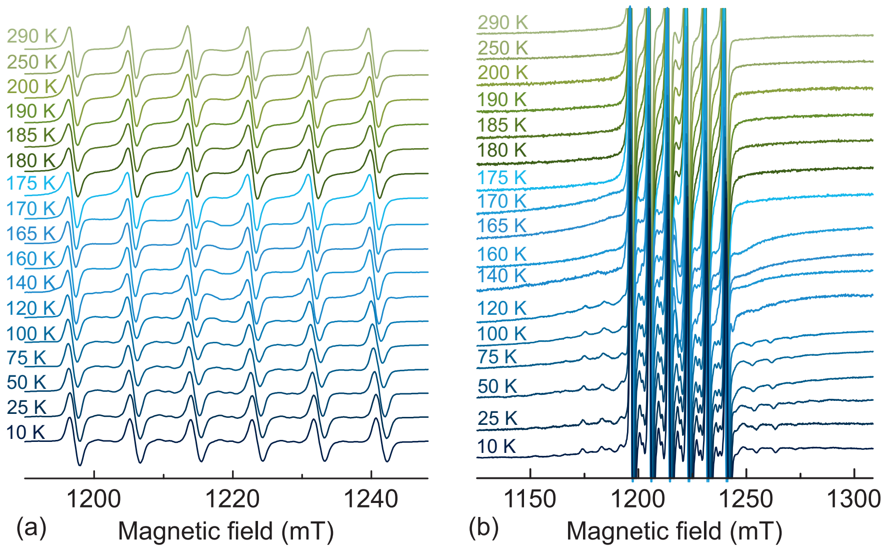

Figure S3: Normalized Q-band EPR spectra of $MAPbCl_3$:Mn recorded at different temperatures. Emphasis on the (a) central and (b) outer transitions.

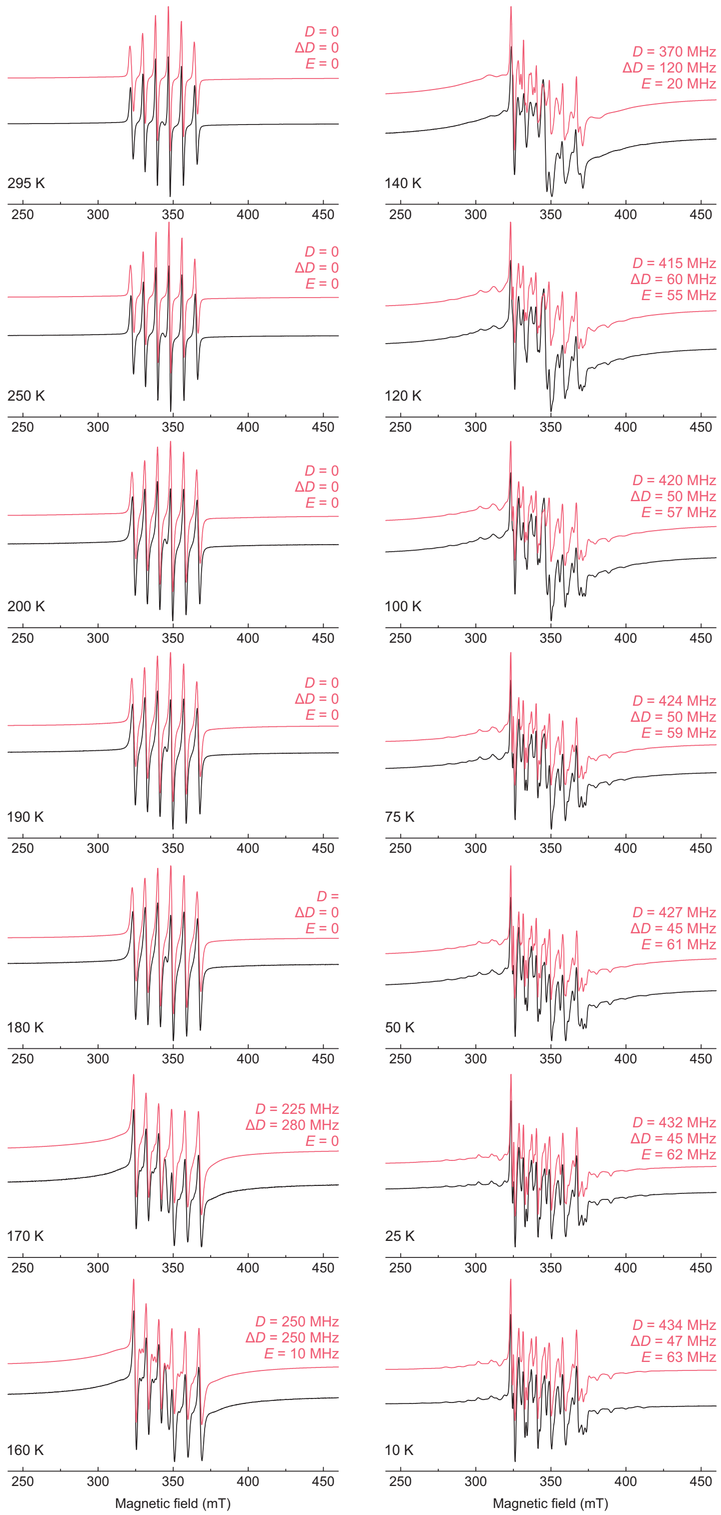

Figure S4: Simulations (red) of the experimental (black) X-band CW EPR spectra of $MAPbCl_3$:Mn obtained at different temperature. Fine structure parameters used for simulations are indicated above the simulated spectra. The simulations also include a broad background line assigned to the clustered Mn^{2+} species.

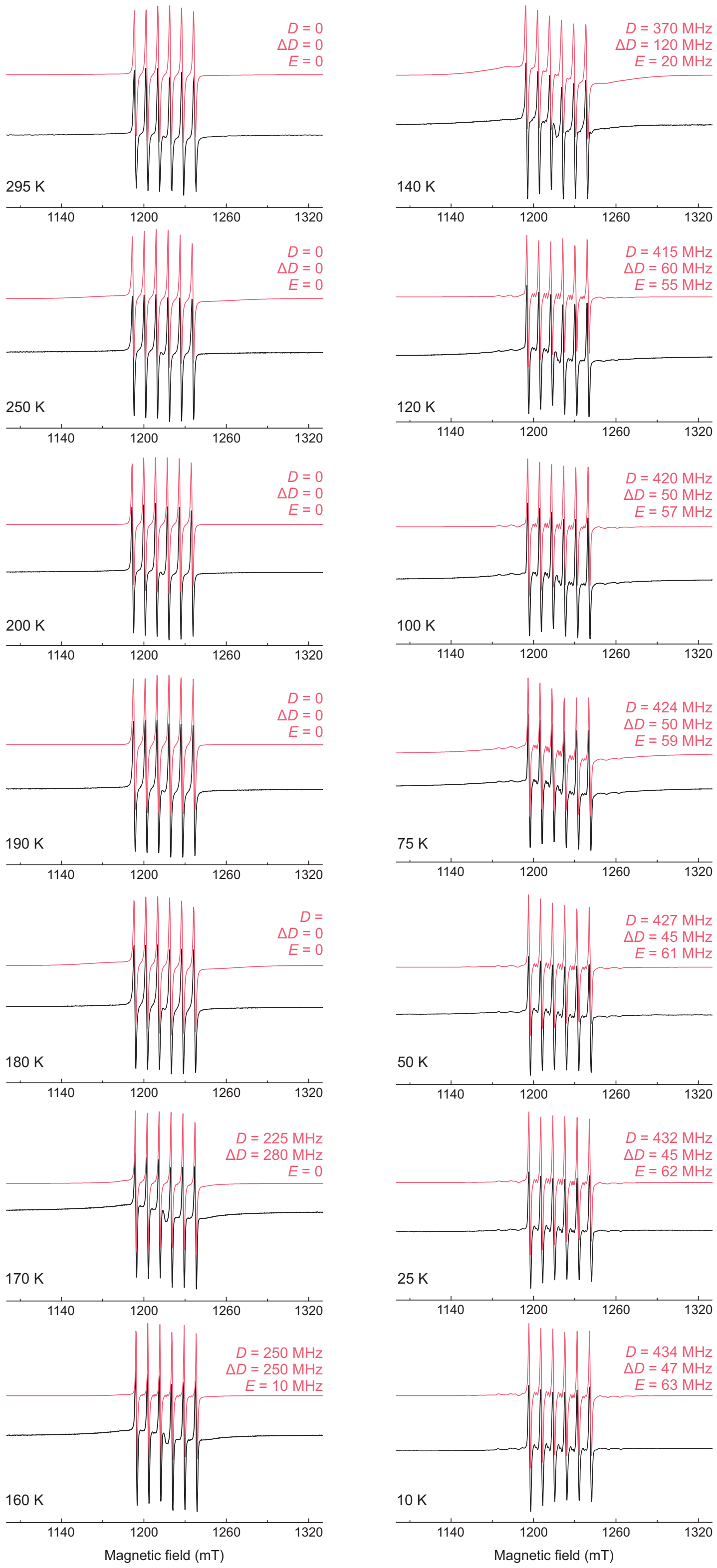

Figure S5: Simulations (red) of the experimental (black) Q-band CW EPR spectra of $MAPbCl_3$:Mn obtained at different temperature. Fine structure parameters used for simulations are indicated above the simulated spectra. The simulations also include a broad background line assigned to the clustered Mn^{2+} species.

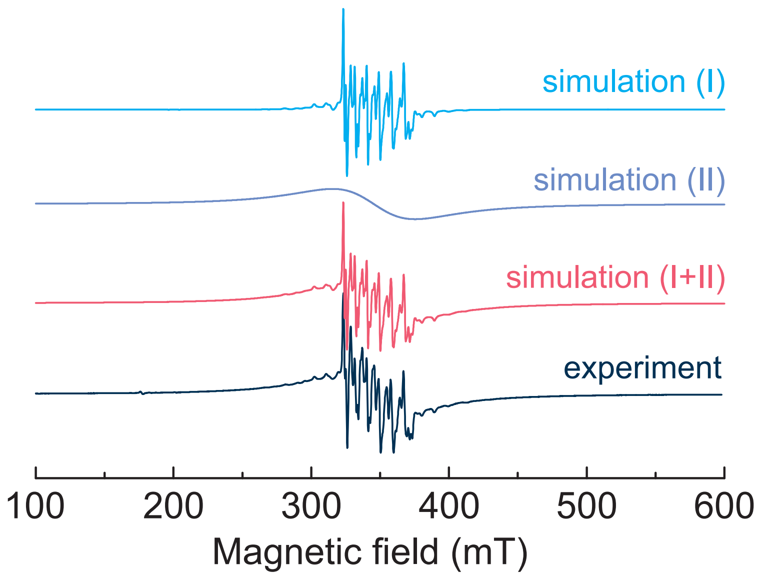

Figure S6: Simulation of the X-band CW EPR spectrum of $MAPbCl_3$:Mn recorded at 50 K, where both signals are well visible. The best agreement with the experiment is obtained by adding a broad EPR line (II) to the Mn^{2+} spectrum (I). The intensity of the broad line is about 10× higher compared to the intensity of the well-resolved Mn^{2+} spectrum.

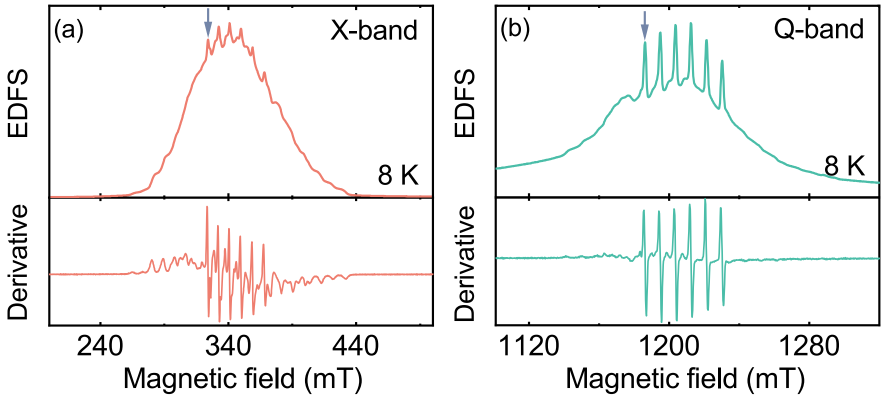

Figure S7: (a) X- and (b) Q-band EDFS spectra of $MAPbCl_3$:Mn obtained at 8 K. The corresponding first derivatives of the spectra are also presented. Arrows indicate the field position for the pulsed EPR experiments.

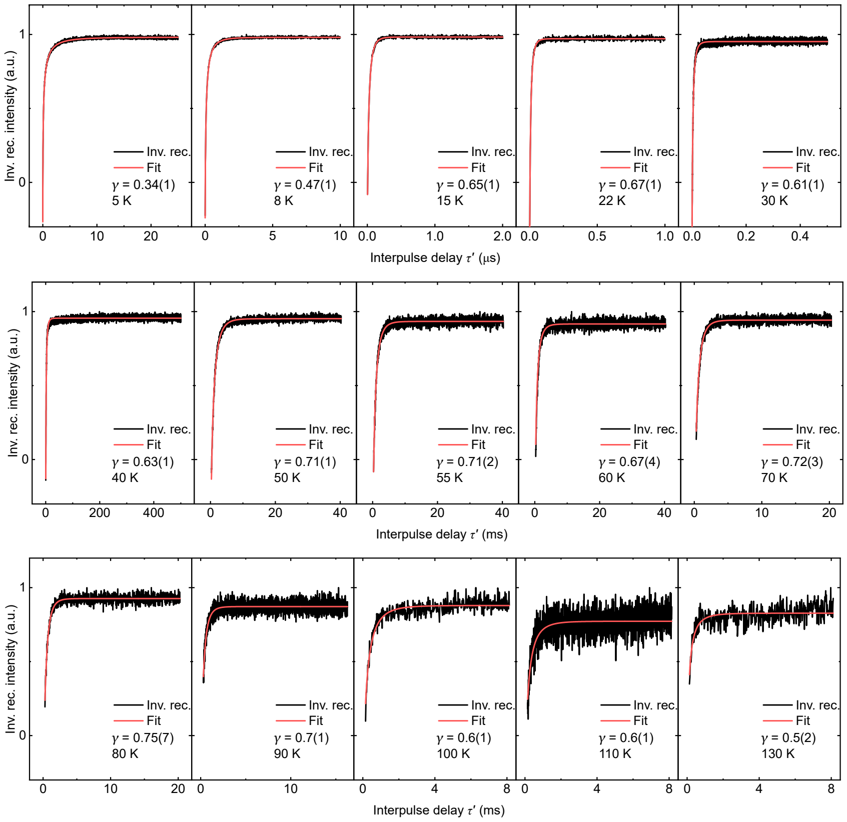

Figure S8: Inversion recovery data of $MAPbCl_3$:Mn obtained at X-band (324.9 mT) frequency and different temperatures. Red curves indicate the best fits to a stretched exponential recovery function: $V = a(1 - b\exp(-(\tau'/T_1)^\gamma))$.

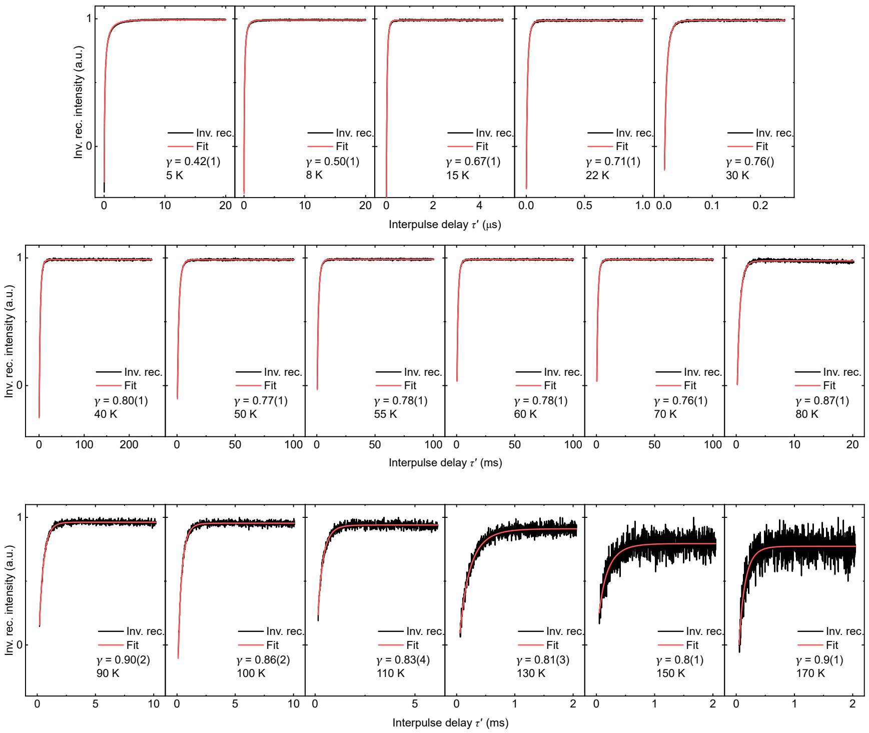

Figure S9: Inversion recovery data of $MAPbCl_3$:Mn obtained at Q-band (1196.7 mT) frequency and different temperatures. Red curves indicate the best fits to a stretched exponential recovery function: $V = a(1 - b\exp(-(\tau'/T_1)^{\gamma}))$.

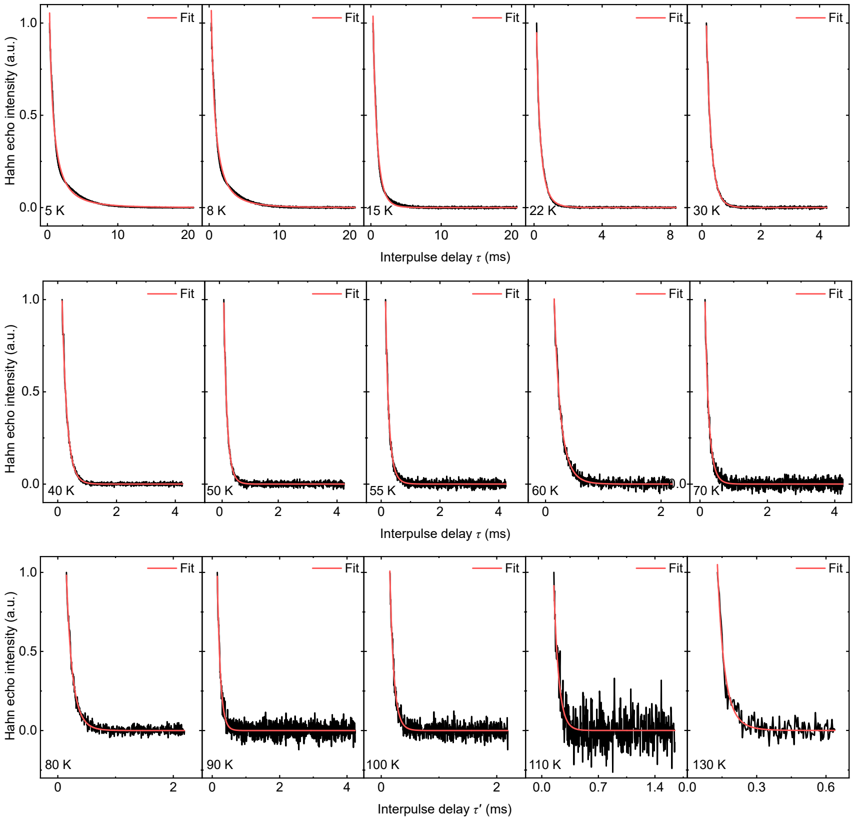

Figure S10: Hahn echo decays of $MAPbCl_3$:Mn obtained at X-band frequency (324.9 mT) and different temperatures. Red curves indicate the best fits to a stretched exponential decay model: $V = a\exp(-(2\tau/T_2)^{\gamma})$.

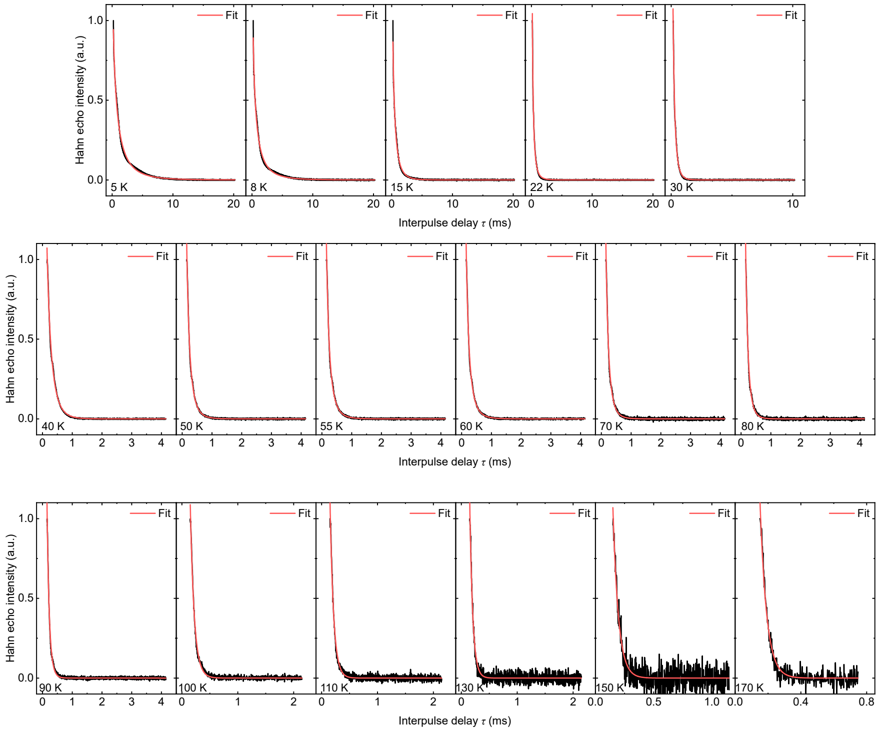

Figure S11: Hahn echo decays of $MAPbCl_3$:Mn obtained at Q-band frequency (1196.7 mT) and different temperatures. Red curves indicate the best fits to a stretched exponential decay model: $V = a\exp(-(2\tau/T_2)^\gamma)$.

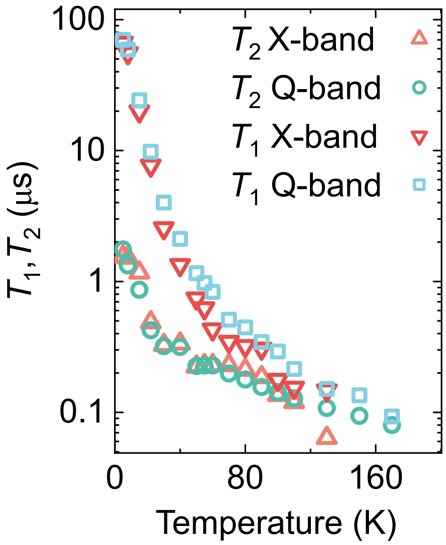

Figure S12: Temperature dependence of T_1 and T_2 times of $MAPbCl_3$:Mn measured at X- and Q-band frequencies.

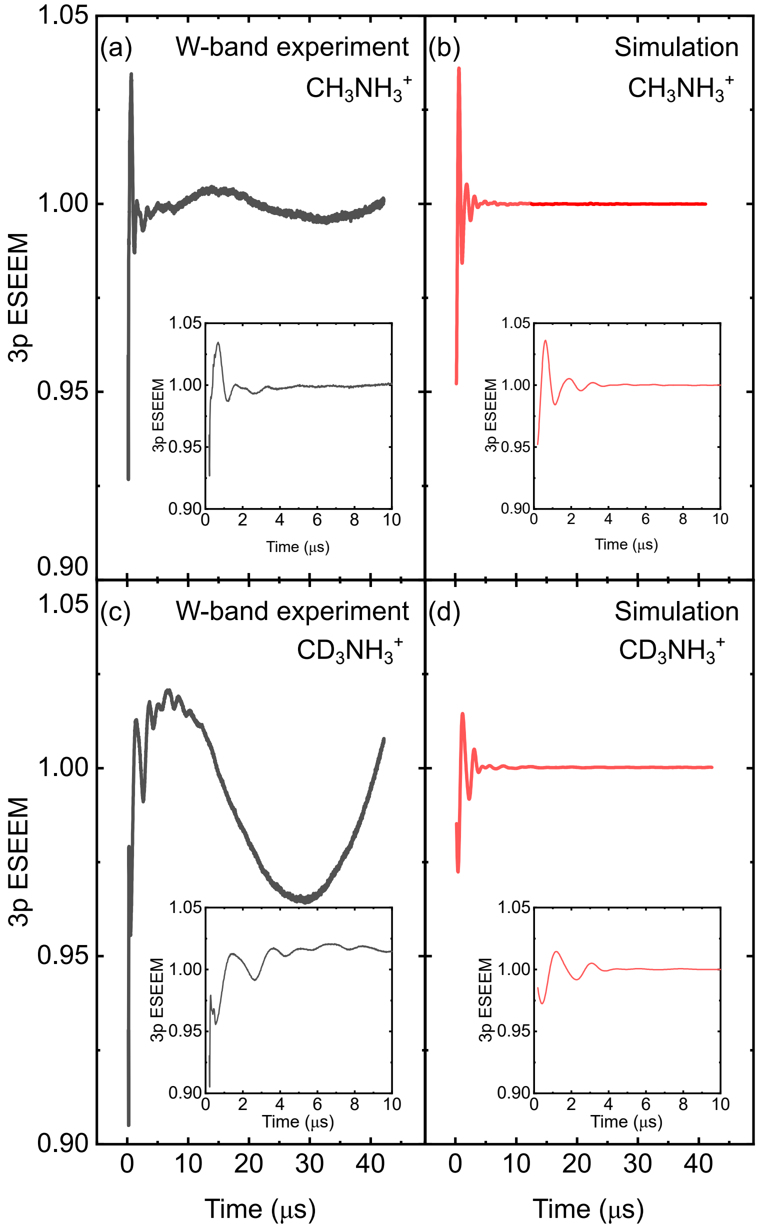

Figure S13: (a,c) Measured (8 K) and (b,d) simulated W-band 3p ESEEM time-domain traces of protonated and partially deuterated $MAPbCl_3$:Mn.

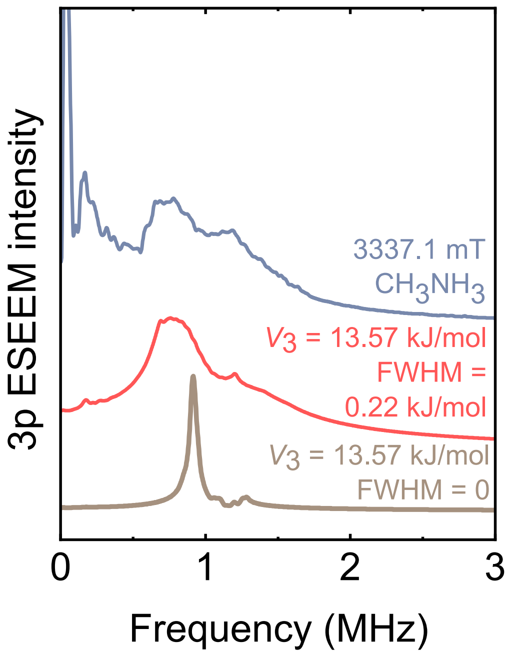

Figure S14: Simulation of the 3p ESEEM spectrum of $MAPbCl_3$:Mn using single-valued ($V_3 = 13.57$ kJ/mol) and distributed ($V_3 = 13.57$ kJ/mol, FWHM = 0.22 kJ/mol) rotational barrier of NH_3 groups. The experimental spectrum is presented for comparison.

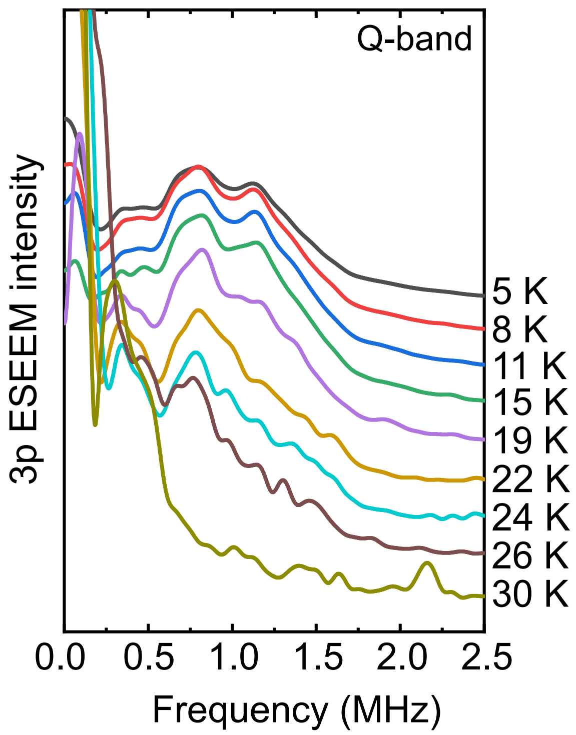

Figure S15: Temperature dependence of the 3p ESEEM spectrum of $MAPbCl_3$:Mn obtained at Q-band (1194.8 mT) frequency.

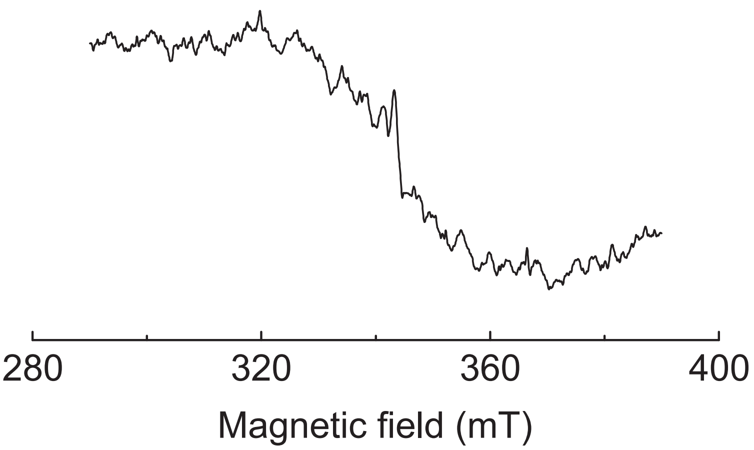

Figure S16: Room-temperature X-band CW EPR spectrum of $MAPbI_3$ mechanosynthesised with 1 mol% $MnBr_2$ showing unsuccessful incorporation of the Mn^{2+} ions.

References

(1) Nandi, P.; Giri, C.; Swain, D.; Manju, U.; Topwal, D. Room Temperature Growth of $CH_3NH_3PbCl_3$ Single Crystals by Solvent Evaporation Method. *CrystEngComm* **2019**, *21*, 656–661.

(2) Mączka, M.; Zienkiewicz, J. A.; Ptak, M. Comparative Studies of Phonon Properties of Three-Dimensional Hybrid Organic–Inorganic Perovskites Comprising Methylhydrazinium, Methylammonium, and Formamidinium Cations. *J. Phys. Chem. C* **2022**, *126*, 4048–4056.
